# MHJ_0125 is an M42 glutamyl aminopeptidase that moonlights as a multifunctional adhesin on the surface of *Mycoplasma hyopneumoniae*

**DOI:** 10.1098/rsob.130017

**Published:** 2013-04

**Authors:** Mark W. Robinson, Kyle A. Buchtmann, Cheryl Jenkins, Jessica L. Tacchi, Benjamin B. A. Raymond, Joyce To, Piklu Roy Chowdhury, Lauren K. Woolley, Maurizio Labbate, Lynne Turnbull, Cynthia B. Whitchurch, Matthew P. Padula, Steven P. Djordjevic

**Affiliations:** 1 The ithree institute, University of Technology, Sydney PO Box 123, Broadway, New South Wales 2007, Australia; 2 Proteomics Core Facility, University of Technology, Sydney PO Box 123, Broadway, New South Wales 2007, Australia; 3 School of Biological Sciences, Queen's University, 97 Lisburn Road, Belfast, Northern Ireland; 4 NSW Department of Primary Industries, PMB 8, Camden, New South Wales 257, Australia

**Keywords:** *Mycoplasma*, aminopeptidase, moonlighting, plasminogen, heparin, homopolymeric complex

## Abstract

Bacterial aminopeptidases play important roles in pathogenesis by providing a source of amino acids from exogenous proteins, destroying host immunological effector peptides and executing posttranslational modification of bacterial and host proteins. We show that MHJ_0125 from the swine respiratory pathogen *Mycoplasma hyopneumoniae* represents a new member of the M42 class of bacterial aminopeptidases. Despite lacking a recognizable signal sequence, MHJ_0125 is detectable on the cell surface by fluorescence microscopy and LC-MS/MS of (i) biotinylated surface proteins captured by avidin chromatography and (ii) peptides released by mild trypsin shaving. Furthermore, surface-associated glutamyl aminopeptidase activity was detected by incubation of live *M. hyopneumoniae* cells with the diagnostic substrate H-Glu-AMC. MHJ_0125 moonlights as a multifunctional adhesin, binding to both heparin and plasminogen. Native proteomics and comparative modelling studies suggest MHJ_0125 forms a dodecameric, homopolymeric structure and provide insight into the positions of key residues that are predicted to interact with heparin and plasminogen. MHJ_0125 is the first aminopeptidase shown to both bind plasminogen and facilitate its activation by tissue plasminogen activator. Plasmin cleaves host extracellular matrix proteins and activates matrix metalloproteases, generating peptide substrates for MHJ_0125 and a source of amino acids for growth of *M. hyopneumoniae*. This unique interaction represents a new paradigm in microbial pathogenesis.

## 2. Introduction

Mucosal droplets released from *Mycoplasma hyopneumoniae*-infected swine during bouts of coughing are important in the cycle of re-infection of naive animals within the confines of a commercial swine production facility. Upon inhalation, *M. hyopneumoniae* is confronted by the mucociliary clearance machinery, a key innate immune barrier to infection. *Mycoplasma hyopneumoniae* must also traverse mucus barriers and initiate colonization by adhering to cilia if infection is to proceed. Ciliostasis, cilial loss and epithelial cell death are pathological features of infection by *M. hyopneumoniae* [1–3]. Infection by *M. hyopneumoniae* stimulates the expression of proinflammatory cytokines such as interleukin-1β, tumour necrosis factor-alpha and interleukin-6 that elicit an acute inflammatory response attracting neutrophils and monocytes to the infected airways [3–5]. Although chronic inflammation is a hallmark of *M. hyopneumoniae* infection, the mechanisms by which *M. hyopneumoniae* initiates cell damage and influences inflammation are not well understood. *Mycoplasma hyopneumoniae* is detectable in the spleen, liver and kidneys of artificially challenged pigs and their cohorts [6–10], but further studies are needed to determine if it plays a pathogenic role at these distal tissue sites. *Mycoplasma hyopneumoniae* must display an elaborate and extensive repertoire of surface antigens to interact with extracellular matrix (ECM) components and diverse cell types.

*Mycoplasma hyopneumoniae* presents members of the P97 and P102 paralogue families of adhesin proteins on the cell surface, where their primary role is to adhere to respiratory cilia. These multifunctional adhesins undergo endoproteolytic processing such that the N-terminal cleavage product retains the signal peptide, and central and C-terminal fragments are released from the preprotein but remain associated with the cell surface [4,11–19]. The pattern of proteolytic cleavage fragments is often consistent among strains derived from different geographical origins, but some fragments are strain specific or are more prominent because the efficiency of cleavage at a specific site may vary [17–19]. Cleavage occurs predominantly at the carboxyl side of phenylalanine residues that reside within the motif S/T-X-F↓-X-D/E [11] but at least two other cleavage motifs have been described [16,17].

Cleavage fragments generated from P97 and P102 paralogue families are multifunctional. Many bind sulfated glycosaminoglycans and fibronectin [11–16,18–20], and these interactions are critical for successful colonization of the respiratory tract [15,21] and porcine epithelial cells [4,14,15,19]. Cleavage fragments also bind plasmin(ogen) [4,12,13,18]. Plasminogen is readily detectable in the fluid lining ciliated epithelial surfaces in the porcine respiratory tract and is sequestered onto the surface of *M. hyopneumoniae. Mycoplasma hyopneumoniae* surface-bound plasminogen is readily converted to the serine protease plasmin by tissue plasminogen activator (tPA) and is capable of degrading fibrinogen [4]. Plasmin levels are consistently elevated in bronchoalveolar lavage (BAL) fluids of pigs infected with *M. hyopneumoniae* compared with BAL fluid collected from the same animals prior to challenge [4]. The recruitment of plasmin(ogen) to the surface of *M. hyopneumoniae* is likely to play an important role in its ability to colonize the respiratory tract, traverse ECM/basement membrane and colonize sites distal to the respiratory tract.

Proteases profoundly influence the surface protein topography of *M. hyopneumoniae* [16,18]. The genomes of *M. hyopneumoniae* strains J, 232 and 7448 are predicted to encode 10 putative proteases including signal peptidase 1 (MHJ_0022, Q4AAS7), lipoprotein signal peptidase (MHJ_0027, Q4AAS2), ATP-dependent zinc metalloprotease FtsH (MHJ_0098, Q4AAC8), heat-shock ATP-dependent ion protease (MHJ_0525, Q4A9G0), putative aminopeptidase (MHJ_0125, Q4AAK4), subtilisin-like serine protease (MHJ_0085, Q4AAP2), methionine aminopeptidase (MHJ_0169, Q4AAG1), leucyl aminopeptidase (MHJ_0461, Q4A9M4), oligoendopeptidase F (MHJ_0522, Q4A9G3) and Xaa-Pro aminopeptidase (MHJ_0659, Q4A929), [22–24]. Our proteome studies, performed on strain J, indicate that eight putative proteases are expressed during broth culture (S. P. Djordjevic and J. L. Tacchi 2010, unpublished results).

In the present study, MHJ_0125 was cloned and expressed as a functionally active polyhistidine fusion (rMHJ_0125) to determine its function*.* We also investigated the subcellular location and three-dimensional structure of MHJ_0125. The biochemical properties of MHJ_0125 and the potential role(s) in pathogenesis of this moonlighting protease are discussed.

## 3. Results

### 3.1. MHJ_0125 resides on the surface of *Mycoplasma hyopneumoniae* and is secreted extracellularly

**Figure 1.**
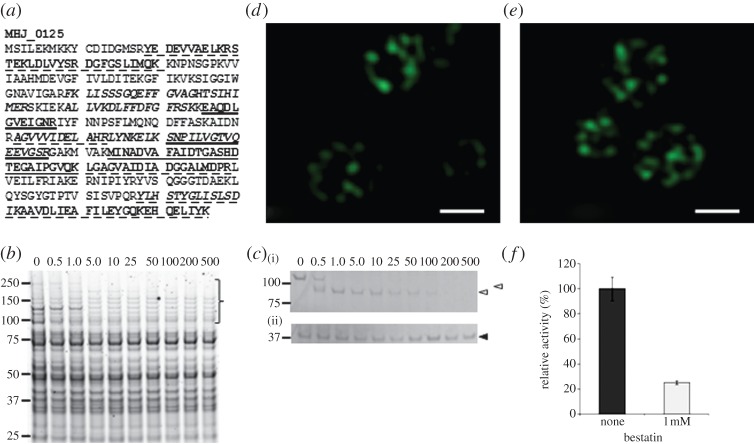
MHJ_0125 is a surface-accessible and secreted protein. (*a*) Tryptic peptides matching to MHJ_0125 from surface proteome analyses. Peptides in italics were identified by shaving freshly cultured *M. hyopneumoniae* cells with trypsin. Peptides underscored with a dashed line correspond to tryptic peptides of biotinylated MHJ_0125 recovered by avidin chromatography. Single underlined peptides were generated by digesting *M. hyopneumoniae* proteins released in PBS (secreteome) with trypsin. Double underscored tryptic peptides were common in preparations derived from avidin chromatography and secreteome studies. (*b*) Cell lysates of *M. hyopneumoniae* cells exposed to different concentrations of trypsin (0–500 µg ml^−1^ at 37°C for 15 min). Protein profiles indicate that the cell membrane remains intact under these experimental conditions. (*c*) Western blot of *M. hyopneumoniae* lysates from cells exposed to different concentrations of trypsin probed with rabbit anti-P97 N-terminal serum (i) and anti-MHJ_0125 serum (ii). The P97 cilium adhesin (top empty arrow) is reported to be highly sensitive to trypsin [17] and is recognized by anti-P97 N-terminal antibodies. MHJ_0125 was not completely degraded by trypsin because of its presence inside the cell cytosol. (*d*,*e*) Structured illumination microscopy images of *M. hyopneumoniae* cells probed with rabbit anti-MHJ_0125 followed by goat anti-rabbit HRP conjugated to Alexa Fluor 488. Fixed, non-permeabilized cells (*d*) show MHJ_0125 only on the cell surface, whereas permeabilized, fixed cells (*e*) show MHJ_0125 is also present inside the cell. Images were taken on an OMX Deltavision microscope. Scale bar, 0.5 µm. (*f*) Cell surface-associated glutamyl aminopeptidase activity was confirmed by culturing live *M. hyopneumoniae* in the presence of the fluorescent substrate H-Glu-AMC, with or without the aminopeptidase inhibitor bestatin. Data are shown as the relative enzyme activity, expressed as a percentage against no bestatin (none) that is taken to be 100% activity. Data represent mean fluorescence units from three independent assays±s.d.

A subset of tryptic peptides detected by LC-MS/MS that were generated by mildly enzymatically shaving the surface of *M. hyopneumoniae* cells mapped to a protein (MHJ_0125, UniProt number Q4AAK4) annotated as a putative aminopeptidase (figure 1*a*). This was a surprising observation because MHJ_0125 lacks a signal peptide, has no apparent transmembrane spanning regions and is predicted to reside intracellularly by PSORTb. In control experiments performed in triplicate, proteins identified by mass spectrometric analyses of tryptic digests of proteins released from *M. hyopneumoniae* incubated under identical conditions used for the shaving, but in the absence of trypsin, identified four proteins. All four proteins belonged to a subset of proteins localized to the cell surface. Tryptic digestion of secreted proteins identified peptides that mapped to MHJ_0125 (figure 1*a*). On no occasion did we observe proteins in comparable concentration as MHJ_0125 as determined by spectral counting, that we failed to identify in our surface studies. These observations indicated that cell lysis was not responsible for detecting MHJ_0125 extracellularly. Consistent with this, we did not observe appreciable cell lysis with incubations over time from 30 s to 30 min by SDS-PAGE (see the electronic supplementary material, figure S1).

To further address the possibility that MHJ_0125 resides on the surface of *M. hyopneumoniae*, surface-accessible proteins were labelled with Sulfo-NHS-LC-Biotin, captured by avidin chromatography and separated by two-dimensional gel electrophoresis. Tryptic peptides mapping to MHJ_0125 were generated from a biotinylated protein spot (data not shown) with a mass of approximately 40 kDa. The MHJ_0125 protein (356 amino acids) has a predicted molecular mass of 39.2 kDa and a theoretical p*I* value of 6.21. These surface proteome studies indicate that MHJ_0125 both resides on the cell surface and is shed into the extracellular milieu.

To determine if MHJ_0125 exists solely on the extracellular side of the membrane, *M. hyopneumoniae* cells were exposed to a range of trypsin concentrations. Cell lysates stained with Flamingo stain (figure 1*b*) showed the gradual proteolysis of proteins with increasing trypsin concentrations, especially in the high mass regions. Many proteins, however, remained unaffected even at the highest trypsin concentration (500 µg ml^−1^), indicating that the cell membrane remained intact. These observations are consistent with previous experiments [14–17]. Cell lysates of shaved cells were blotted onto polyvinylidene fluoride (PVDF) membrane and probed with rabbit anti-MHJ_0125 antibodies. MHJ_0125 was detectable in the control lane (no trypsin) and at all trypsin concentrations up to 500 µg ml^−1^ (figure 1*c*(ii)). Anti-MHJ_0125 antibodies detected a single protein species with a mass of 39 kDa, confirming it is monospecific for MHJ_0125 (see the electronic supplementary material, figure S2). The same blot was re-probed with P97 N-terminal serum that recognizes the central portion of the P97 adhesin [17]. There was clear evidence that P97 was readily digested at a trypsin concentration of 1 µg ml^−1^ (figure 1*c*(i)). These data indicate that MHJ_0125 resides in the cytosol of *M. hyopneumoniae*, but that a subset of MHJ_0125 molecules are both bound to and secreted from the cell surface.

Fluorescence microscopy was also used to examine the cellular location of MHJ_0125. *Mycoplasma hyopneumoniae* cells were stained with anti-MHJ_0125 antibodies and detected using goat anti-rabbit antibodies conjugated with Alexa Fluor 488. Widefield conventional fluorescence microscopy suggested that all *M. hyopneumoniae* cells produced a fluorescent ring surrounding the cell surface (electronic supplementary material, figure S3*a*). When *M. hyopneumoniae* cells were pretreated with Triton X-100 to permeabilize the *Mycoplasma* membranes, the fluorescence pattern instead filled the entire cell body and the fluorescence intensity was brighter compared with images generated with non-permeabilized cells imaged at the same intensity (see the electronic supplementary material, figure S3*b*). In control experiments, goat anti-rabbit Alexa Fluor antibodies did not contribute to staining of *M. hyopneumoniae* cells in the absence of anti-MHJ_0125 antibodies (data not shown). To further confirm the subcellular localization of the MHJ_0125 proteins, the cells were examined using super-resolution three-dimensional structured illumination microscopy (3D-SIM). These studies confirmed that MHJ_0125 resides both on the surface (figure 1*d*) and in the cytosol (figure 1*e*) of all *M. hyopneumoniae* cells growing *in vitro*.

### 3.2. MHJ_0125 forms a homopolymeric complex

**Figure 2.**
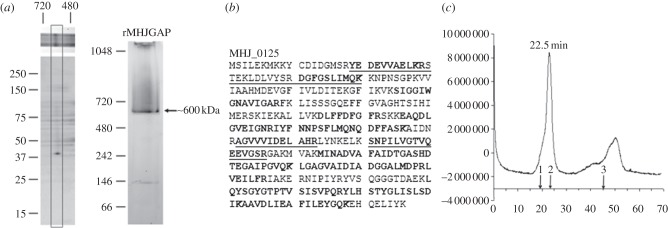
Native and rMHJ_0125 form homopolymeric complexes. (*a*) Analysis of *M. hyopneumoniae* soluble proteins by native PAGE (top). Further separation of the 600 kDa band by two-dimensional native/SDS-PAGE (bottom) resolved a single-protein spot with an approximate molecular mass of 40 kDa. LC-MS/MS confirmed that the spot was MHJ_0125. Analysis of purified recombinant MHJ_0125 by one-dimensional native PAGE confirmed that it forms an approximately 600 kDa complex. (*b*) In-gel trypsin digestion and LC-MS/MS of tryptic peptides generated from the 600 kDa band (matches in bold), and the corresponding 40 kDa spot (matches underlined) matched to MHJ_0125. (*c*) Size exclusion chromatography of rMHJ_0125 through Superdex 200. Arrows indicate the elution position of commercial size markers blue dextran (2000 kDa), apoferritin (443 kDa) and myoglobin (17 kDa). rMHJ_0125 migrates at a mass of approximately 440 kDa.

Analysis of *M. hyopneumoniae* cell lysates by native PAGE identified a prominent high molecular mass band of approximately 600 kDa that resolved as a single-protein spot with a mass of about 40 kDa, following separation by denaturing SDS-PAGE in the second dimension (figure 2*a*). Recombinant MHJ_0125 expressed in *Escherichia coli* (rMHJ_0125) also migrates as an approximately 600 kDa band during native PAGE. LC-MS/MS of tryptic peptides generated from the 40 kDa spot mapped exclusively to MHJ_0125 (figure 2*b*). Native PAGE is not a reliable method for accurately predicting the size of protein complexes. Size exclusion chromatography determined that rMHJ_0125 has a mass of approximately 440 kDa under native conditions (figure 2*c*), consistent with the molecule forming a dodecameric complex similar to other M42 glutamyl aminopeptidases [25].

### 3.3. Biochemical characterization of functionally active recombinant MHJ_0125

**Figure 3.**
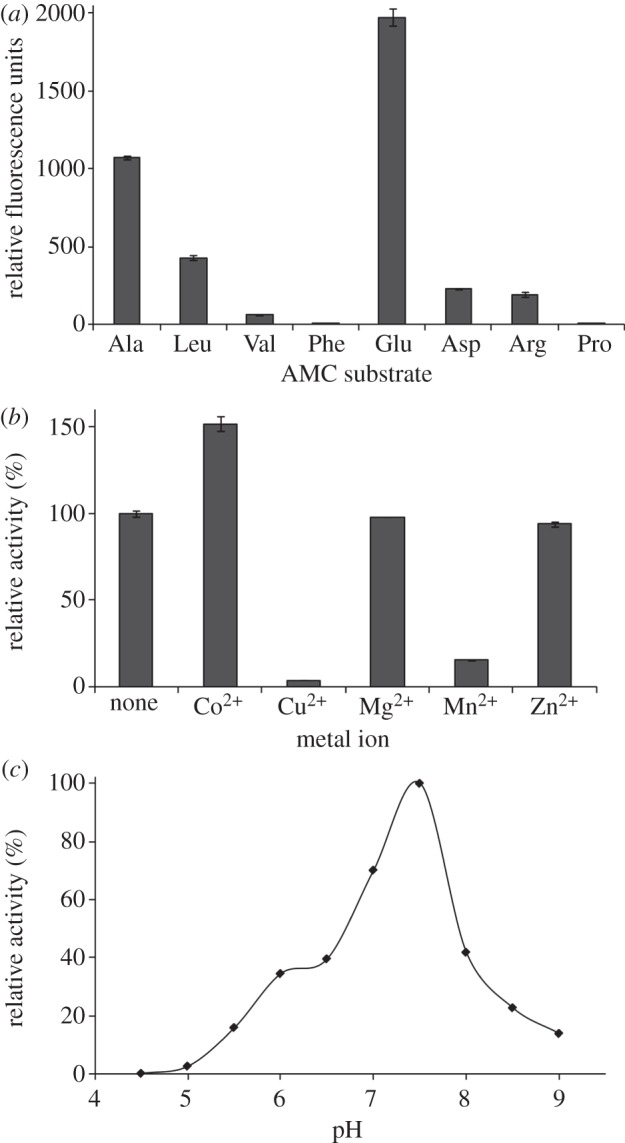
Biochemical characterization of MHJ_0125. (*a*) The relative activity of recombinant M42 glutamyl aminopeptidase against a panel of fluorescent substrates was determined by monitoring the release of the fluorogenic leaving group (-AMC) over 1 h at 37°C. Data represent the mean activity (fluorescence units) of three independent assays±s.d. (*b*) Effect of divalent metal ions on the hydrolysis of H-Glu-AMC by recombinant *M. hyopneumoniae* M42 glutamyl aminopeptidase. Data are shown as the relative enzyme activity, expressed as a percentage against no metal ion (none) that is taken to be 100% activity. Data represent mean fluorescence units from three independent assays±s.d. (*c*) Initial rates of hydrolysis of H-Glu-AMC by recombinant *M. hyopneumoniae* M42 glutamyl aminopeptidase were measured over 1 h in a variety of buffers (pH 4.5–9.0). The enzyme exhibits greatest activity in the pH range 6.0–8.5, with a pH optimum at 7.5.

Recombinant MHJ_0125 was purified from *E. coli* cell lysates by nickel affinity chromatography. A protein of approximately 42 kDa was resolved by SDS-PAGE and its identity was confirmed as rMHJ_0125 by LC-MS/MS analysis (data not shown). rMHJ_0125 efficiently cleaves the acidic amino acid glutamic acid from the N-terminus of synthetic fluorogenic peptide substrates (figure 3*a*) but not aspartic acid which, unexpectedly, it cleaved relatively poorly. Interestingly, rMHJ_0125 also cleaved H-Ala-AMC and to a lesser extent H-Leu-AMC (figure 3*a*). Very limited or no hydrolysis was observed against the fluorogenic substrates H-Arg-AMC, H-Pro-AMC, H-Val-AMC and H-Phe-AMC. These data are consistent with the classification of MHJ_0125 as a member of the M42 glutamyl aminopeptidase family. One of the best characterized bacterial M42 glutamyl aminopeptidases is *Streptococcus pneumoniae* PepA (SpPepA) [25]. A comparison of the substrate specificity of MHJ_0125 with that of SpPepA showed that while both enzymes have a strong preference for glutamic acid, they differ in their ability to cleave aspartic acid residues; SpPepA shows 70 per cent relative activity towards aspartic acid [25] compared with 54 per cent shown by MHJ_0125. Both enzymes were also able to cleave alanine residues to some extent (approx. 20% relative activity by SpPepA [25] and 54% by MHJ_0125). The activity of both enzymes against substrates containing other amino acids was negligible. These differences in substrate specificity may be due to variation within the S1 substrate-binding pocket (see §3.8).

rMHJ_0125 activity was reduced to 57.1, 30.1 and 13.3 per cent after incubation with 0.01, 0.1 and 1.0 mM bestatin, respectively. Similarly, rMHJ_0125 activity was reduced to 3.7 per cent following incubation with 10 mM EDTA, demonstrating that metal ions are necessary for enzyme activity (table 1). A study of the effect of various divalent metal ions (final concentration 5 mM) on the activity of the enzyme (against H-Glu-AMC) showed that it was enhanced by Co^2+^ (52%). By contrast, Mn^2+^ considerably reduced enzyme activity (by 84.5%), whereas Cu^2+^ (5 mM) almost abolished enzyme activity (96.5% reduction). Mg^2+^ and Zn^2+^ did not appreciably affect enzyme activity compared with control assays where no metal ions were added (figure 3*b*). The purified enzyme exhibited aminopeptidase activity against H-Glu-AMC between pH 6.0 and 8.5 with optimal activity at pH 7.5 (figure 3*c*). Activity against the fluorogenic substrate H-Glu-AMC was detected with freshly cultured, washed *M. hyopneumoniae* cells, confirming the presence of surface-associated glutamyl aminopeptidase activity. This activity was inhibited *in situ* by 1 mM bestatin (figure 1*f*).

**Table 1.** Effect of the aminopeptidase inhibitor bestatin and the metal chelator EDTA on recombinant *M. hyopneumoniae* M42 glutamyl aminopeptidase activity. Data represent the mean relative activity of three independent assays±s.d.

reagent	concentration (mM)	relative activity (%)
none		100
bestatin	1	13.3 ± 4
	0.1	30.1 ± 5
	0.01	57.1 ± 3
EDTA	10	3.7 ± 3
	1	96.7 ± 7
	0.1	98.3 ± 6

### 3.4. MHJ_0125 binds and activates porcine plasminogen

**Figure 4.**
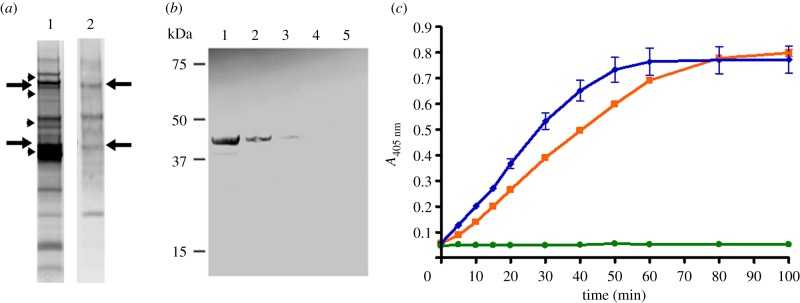
MHJ_0125 binds host plasminogen. (*a*) SDS-PAGE of *M. hyopneumoniae* proteins with approximately p*I* 6 that were fractionated using a MicroRotofor and stained with Flamingo (lane 1). Corresponding proteins blotted onto PVDF and reacted with biotinylated porcine plasminogen (lane 2). Proteins marked with an arrow in lane 1 were cut from the gel and treated for LC-MS/MS. Trypic peptides matched to MHJ_0125 on both occasions. Arrowheads depict the position of molecular mass markers. Marker sizes (arrowheads) in lane 1 are 100, 75, 50 and 37 kDa. (*b*) Ligand blot of purified rMHJ_0125 probed with biotinylated porcine plasminogen. Lane 1, 1 µg rMHJ_0125; lane 2, 0.5 µg rMHJ_0125; lane 3, 0.25 µg rMHJ_0125; lane 4, 0.125 µg rMHJ_0125; lane 5, 0.0625 µg rMHJ_0125. (*c*) rMHJ_0125 activates plasminogen in the presence of tPA. An increase in the rate of plasminogen activation was observed in the presence of tPA and an 8 : 1 molar excess of rMHJ_0125 (orange, squares) compared to plasminogen and tPA alone (blue, diamonds). Plasminogen activation was also enhanced in the presence of a 4 : 1 molar excess of rMHJ_0125 and with tPA but there was no observed increase when plasminogen and rMHJ_0125 were incubated in equimolar ratios with tPA (data not shown). rMHJ_0125 cannot itself cleave plasminogen to plasmin as indicated by incubation of an 8 : 1 molar ratio of rMHJ_0125 with plasminogen in the absence of tPA (green, circles). Data shown are from duplicate samples.

The presence of MHJ_0125 on the surface of *M. hyopneumoniae* suggested that it may function as a virulence factor. Our previous observations showing plasmin activity is elevated in the BAL fluid of pigs infected with *M. hyopneumoniae* [4] and that C-terminal lysines and arginine residues on endoproteolytic cleavage fragments of the P97 and P102 adhesin families bind plasminogen and facilitate its activation to plasmin [12,13,18] led us to investigate whether MHJ_0125 plays a role in plasminogen binding. Consistent with such a role, MHJ_0125 possesses a C-terminal lysine residue. While undertaking a systematic two-dimensional ligand blotting study of *M. hyopneumoniae* plasminogen-binding proteins, we identified two proteins approximately 40 and 80 kDa in size that bound biotinylated plasminogen in the Triton X-114 aqueous phase fraction (figure 4*a*). LC-MS/MS of tryptic fragments of these proteins identified MHJ_0125 (Mascot scores of 650 and 120; peptide coverage of 50% and 25%, respectively). We subsequently found that rMHJ_0125 bound to purified porcine plasminogen in a concentration-dependent manner (figure 4*b*). In the presence of tPA, there was a clear increase in the rate of plasmin activity (figure 4*c*) when there was a molar excess (4 : 1 and 8 : 1) of rMHJ_0125 relative to plasminogen; however, rMHJ_0125 did not activate plasminogen in the absence of tPA. These results suggest that binding of MHJ_0125 to plasminogen results in a conformational change leading to enhanced plasmin activity; however, MHJ_0125 does not itself cleave plasminogen to plasmin.

### 3.5. MHJ_0125 binds heparin

**Figure 5.**
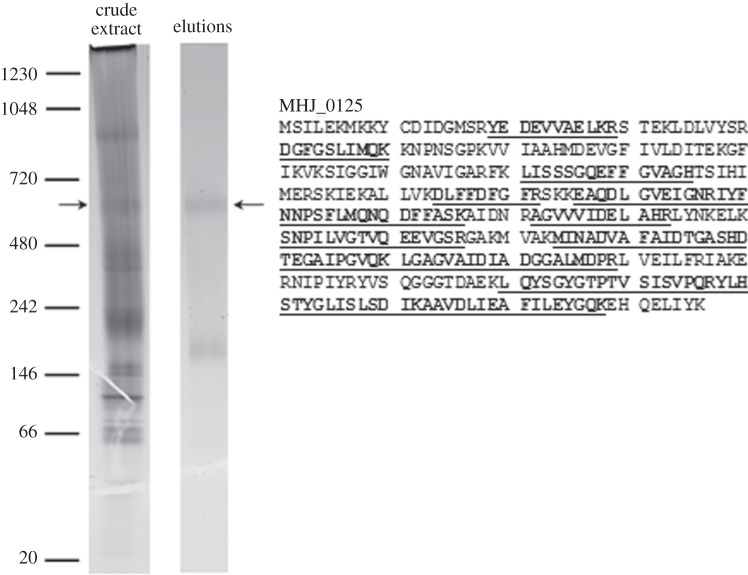
MHJ_0125 binds heparin**.** Native PAGE of *M. hyopneumoniae* proteins that were loaded onto a heparin–agarose column (left lane) and proteins retained on the heparin column. Arrow depicts MHJ_0125 that was identified by LC-MS/MS (boxed sequence with tryptic peptides underpinned).

Heparin is used widely to mimic highly sulfated regions present in glycosaminoglycans and effectively blocks the binding of *M. hyopneumoniae* to porcine cilia [26]. *Mycoplasma hyopneumoniae* binds heparin on its cell surface in a dose-dependent and saturable manner [19]. MHJ_0125 carries a lysine-rich motif (EKMKKY) similar to the consensus heparin-binding site (XBBXBX) at amino acid positions 5–10 in its N-terminus. LC-MS/MS analysis of heparin-binding proteins identified tryptic peptides that mapped to MHJ_0125 (Mascot score 541, 7 unique peptides identified, 28% coverage; data not shown). We had previously determined that MHJ_0125 migrates as a complex with a nominal mass of 600 kDa during native PAGE (figure 2). To determine if this aminopeptidase complex bound heparin, proteins that were retained on the heparin column were separated by one-dimensional native PAGE. LC-MS/MS analysis of tryptic fragments (Mascot score 1277, 14 unique peptides, 49% coverage) generated from the 600 kDa protein complex matched to MHJ_0125 (figure 5).

### 3.6. MHJ_0125 is recognized by convalescent swine antibodies

**Figure 6.**
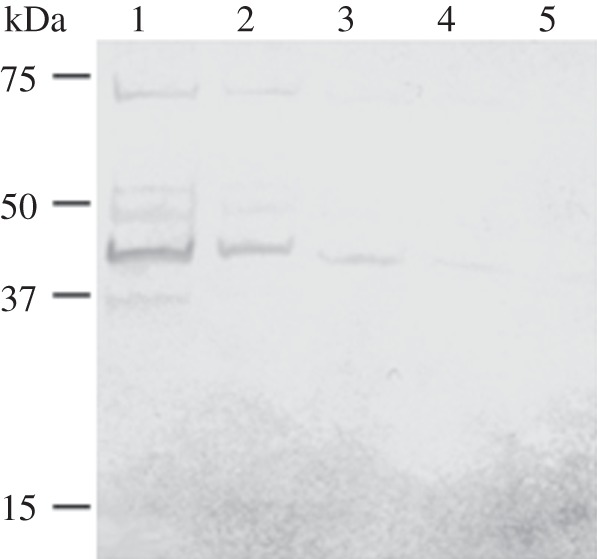
Immunogenicity of MHJ_0125 in *M. hyopneumoniae*-infected, commercially-raised swine. Western blot of recombinant MHJ_0125 probed with convalescent pig sera demonstrating a specific antibody response against the protein. Lane 1, 1 µg rMHJ_0125; lane 2, 0.5 µg rMHJ_0125; lane 3, 0.25 µg rMHJ_0125; lane 4, 0.125 µg rMHJ_0125; lane 5, 0.0625 µg rMHJ_0125.

An antibody response against MHJ_0125 was detected in the serum of commercially reared pigs determined to be infected with *M. hyopneumoniae* by ELISA and western blot (figure 6).

### 3.7. MHJ_0125 is related to other bacterial aminopeptidases

**Figure 7.**
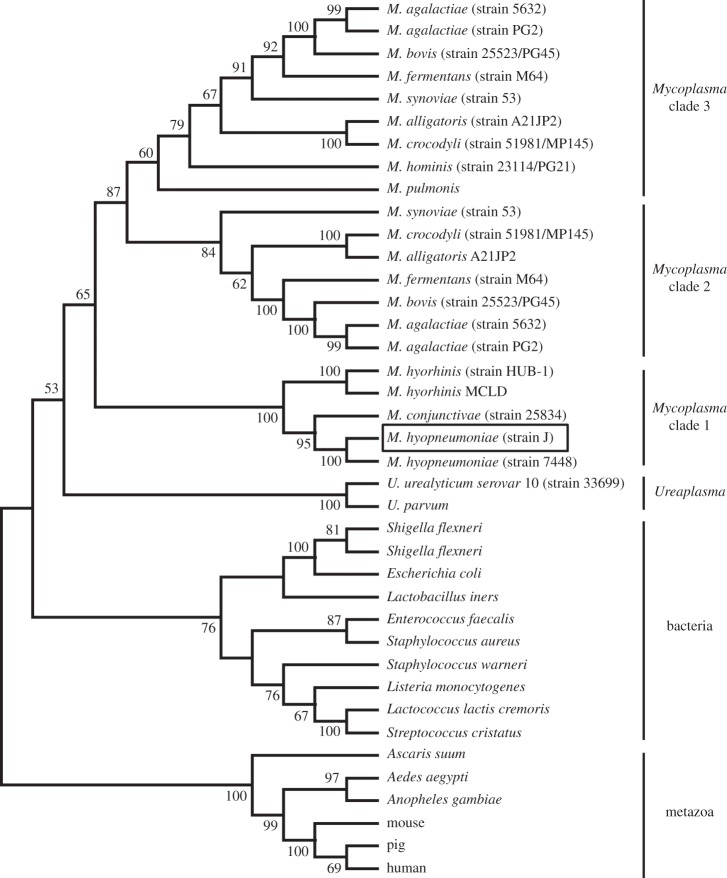
Bootstrapped (1000 trials) neighbour-joining phylogenetic tree showing the evolutionary relationships of the glutamyl aminopeptidases. Numbers represent bootstrap values (given as percentages) for a particular node, and values greater than 50% are shown. The *M. hyopneumoniae* M42 glutamyl aminopeptidase characterized in this study is boxed.

**Figure 8.**
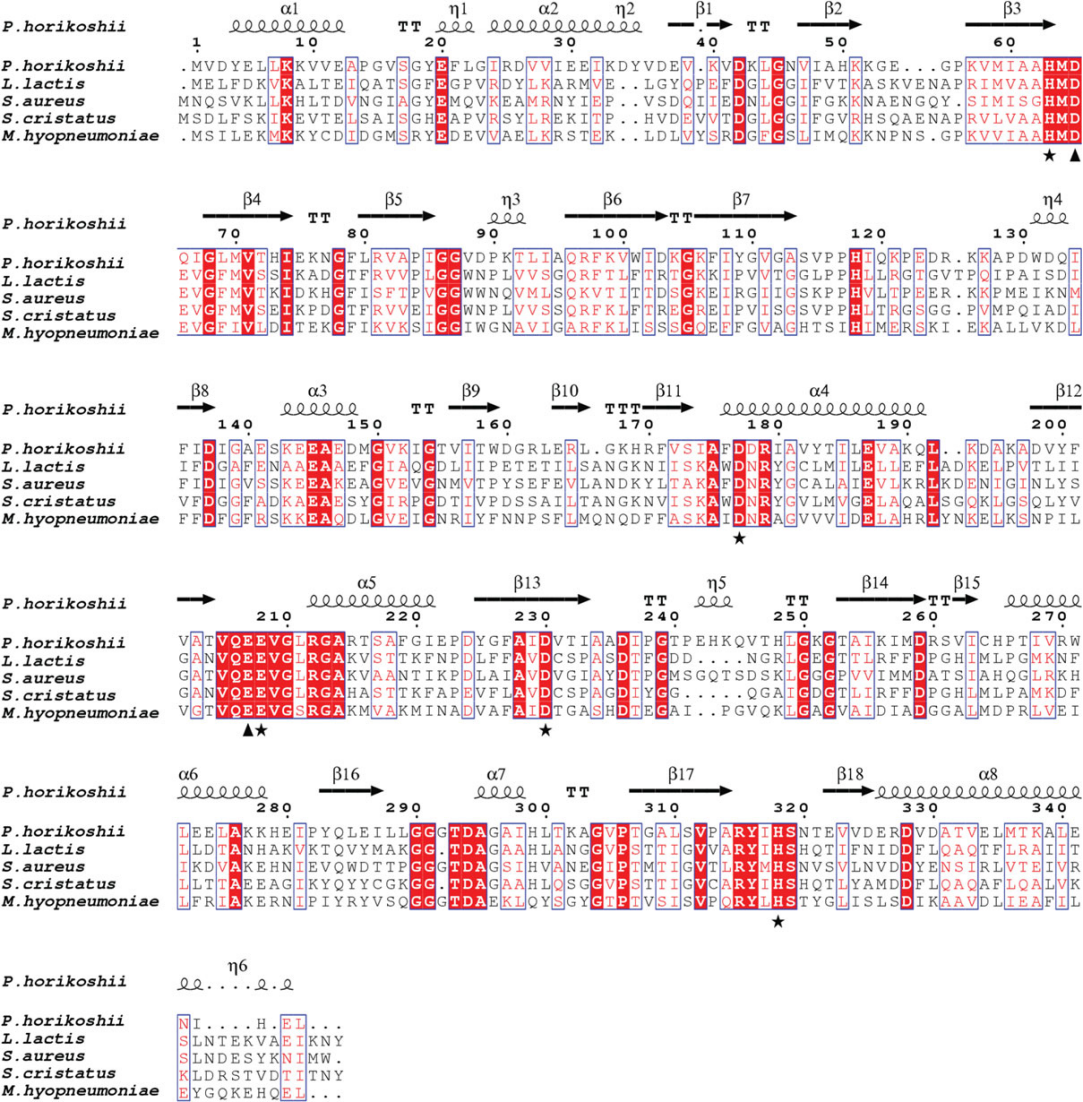
Alignment of M42 glutamyl aminopeptidases from *Mycoplasma hyopneumoniae* (accession number Q4AAK4), *Pyrococcus horikoshii* (accession number NP_143387), *Streptococcus cristatus* (accession number E8JVQ8), *Staphylococcus aureus* (accession number F9K1W5) and *Lactococcus lactis* (accession number Q48677). Identical residues are highlighted in red and conservatively substituted amino acids are in red text. Amino acids involved in metal ion binding (His^64^, Asp^179^, Glu^212^, Asp^234^ and His^320^; *M. hyopneumoniae* numbering) are shown by asterisks and the catalytic residues (Asp^66^ and Glu^211^; *M. hyopneumoniae* numbering) are indicated with arrowheads. Secondary structure elements derived from the crystal structure of *P. horikoshii* TET protease that were used to align MHJ_0125 and TET protease for comparative modelling are displayed on the top row of the alignment.

We assessed the phylogenetic relationship between the *M. hyopneumoniae* sequence and glutamyl aminopeptidases from bacterial and eukaryotic species. Figure 7 shows that MHJ_0125 is a member of a distinct clade (termed *Mycoplasma* clade 1) with sequences from *Mycoplasma hyorhinis*, *Mycoplasma conjunctivae* as well as a sequence from another *M. hyopneumoniae* strain. Related sequences from a range of other *Mycoplasma* species formed two further distinct clades (*Mycoplasma* clades 2 and 3), whereas the *Ureaplasma* sequences were segregated between *Mycoplasma* clade 1 and the bacterial clade. The conceptually translated MHJ_0125 gene showed identity to M42 glutamyl aminopeptidases from a range of bacteria including *Lactococcus lactis* (31%), *Streptococcus cristatus* (31%) and *Staphylococcus aureus* (30%; figure 8). Primary sequence alignments showed that MHJ_0125 contained the conserved catalytic dyad residues Asp^66^ and Glu^211^ as well as conserved residues involved in metal ion binding (His^64^, Asp^179^, Glu^212^, Asp^234^ and His^320^). Based on these sequence similarities, and the presence of conserved active site residues, we have confirmed that the *M. hyopneumoniae* MHJ_0125 gene encodes a new member of the bacterial M42 glutamyl aminopeptidase family.

### 3.8. Molecular modelling of MHJ_0125 complements the biochemical studies

**Figure 9.**
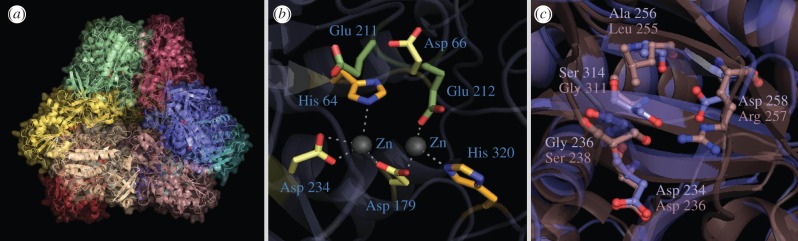
Molecular modelling of MHJ_0125. (*a*) A cartoon rendering of a comparative model of the MHJ_0125 dodecamer based on the tetrahedral crystal structure of *P. horikoshii* TET protease. Individual subunits are coloured. (*b*) A stick representation of the MHJ_0125 active site displaying residues involved in co-ordination of the metal ions (Zn^2+^ in this case) and substrate catalysis. Two zinc ions (Zn^2+^) per subunit have been modelled into the active sites of the complex (red spheres). (*c*) Structure of the substrate-binding (S1) pocket of MHJ_0125 (blue) overlaid with the S1 pocket of PepA glutamyl aminopeptidase from *S. pneumoniae.* PepA contains an arginine residue (Arg^257^) that confers a positive charge on one end of the S1 pocket that is believed to position adjacent to the carboxylate group of the glutamic acid substrate. Curiously, this residue is substituted for Asp^258^ in MHJ_0125; however, it appears that the side chain does not protrude far enough into the S1 pocket to confer a negative charge at this position.

The program Modeller identified tetrahedral aminopeptidase (TET) protease from *Pyrococcus horikoshii* as the most suitable template for construction of the MHJ_0125 comparative model. The secondary structure features used to align MHJ_0125 with *P. horikoshii* TET protease (1Y0R) are presented in figure 9. Because size exclusion chromatography indicated that the MHJ_0125 biological unit is approximately 440 kDa, and given that this protein displays homology to 12-subunit proteases (including TET protease), we reasoned that MHJ_0125 forms a dodecameric complex. Therefore, the comparative model generated for MHJ_0125 contains 12 identical subunits with tetrahedral symmetry based on the biological unit of *P. horikoshii* TET protease (figure 9*a*). Given that the protein databank (PDB) coordinates for TET protease contain zinc in the metal-binding site, and that rMHJ_0125 displays enzymatic activity in the presence of zinc, two Zn^2+^ ions per monomer (24 Zn^2+^ ions in total) were modelled into the active sites of the MHJ_0125 dodecamer (figure 9*a*). Analysis of the quality of the structure with ProSA indicated that the comparative model of MHJ_0125 is in the middle of the range for protein structures determined by X-ray crystallography (*z*-score = −7.24) (see the electronic supplementary material, figure S4).

Our MHJ_0125 model indicates that the conserved residues identified in the sequence alignment (figure 9*b*), which are involved in catalysis and the co-ordination of the metal ions in other aminopeptidases, are in the correct steric position to fulfil these functions in MHJ_0125 (figure 9*b*). This suggests that MHJ_0125 has a similar catalytic mechanism to other aminopeptidases within the superfamily. Interestingly, the substrate-binding pocket of MHJ_0125 differs somewhat from that of the PepA aminopeptidase from *S. pneumoniae* despite the fact that they both display a substrate preference for glutamic acid. Sp PepA contains an arginine residue at position 257 (Arg^257^) that confers a positive charge on the S1 pocket of the binding site that is believed to be juxtaposed to the negatively charged carboxylate group of glutamic acid. In MHJ_0125, an aspartic acid residue (Asp^258^) replaces Arg^257^; however, the carboxyl group of Asp^258^ appears not to protrude far enough into the S1 pocket to confer a negative charge on this region (figure 9*c*). It is probable that this variation in S1 subsite topography is responsible for the minor differences in substrate preference exhibited by SpPepA and MHJ_0125; however, this awaits confirmation via further structure–function analyses.

**Figure 10.**
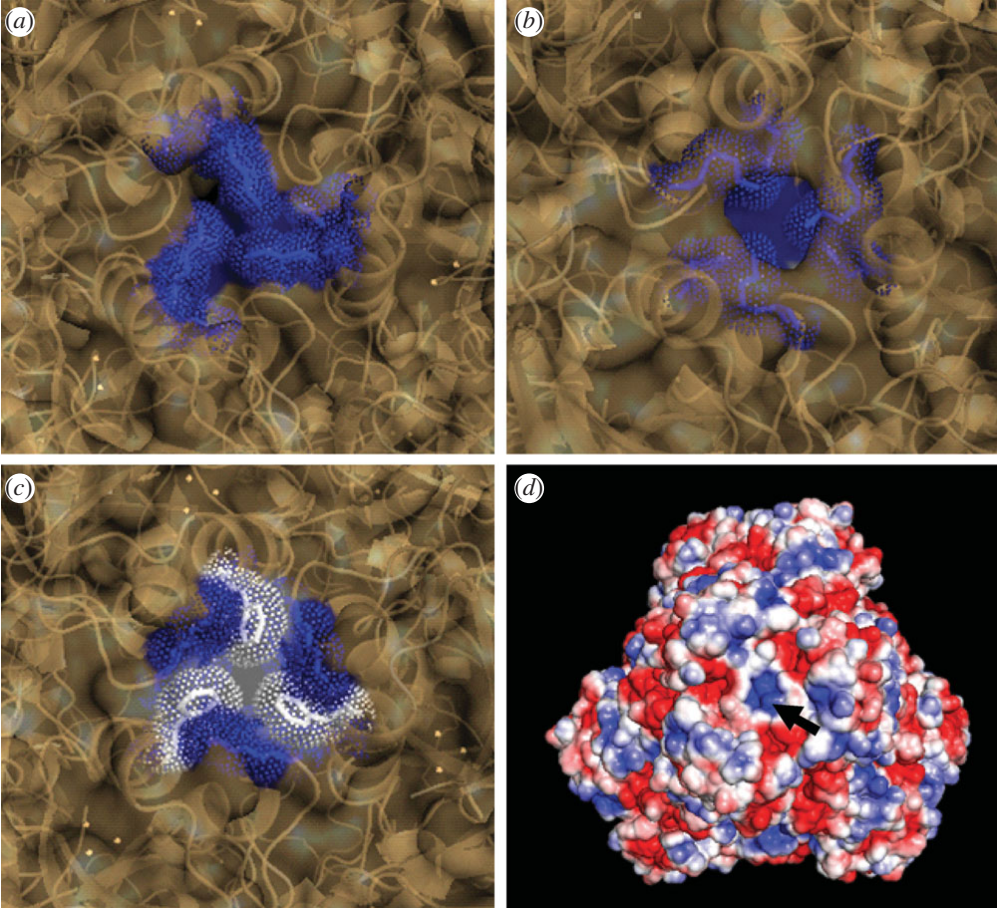
Enlarged view of one of the four small exit pores of (*a*) MHJ_0125, (*b*) *S. pneumoniae* PepA and (*c*) *P. horikoshii* TET protease. Consistent with the substrate preference of these glutamyl aminopeptidases which would mainly export negatively charged glutamic acid residues, the opening of the small pore in (*a*) and (*b*) is dominated by positively charged residues. In contrast and consistent with predominantly leucyl aminopeptidase activity, the TET protease small pore is dominated by 3 hydrophobic phenylalanine residues. The intensely positively charged nature of the MHJ_0125 small pore is highlighted in a rendering of the dodecamer surface overlaid with its surface potential and rotated to look down the axis of the small pore (*d*, arrow).

Like other TETs, MHJ_0125 is predicted to contain four large pores that are entry points for the substrate and four small pores that act as exit channels for the products of catalysis. In MHJ_0125, the large and small openings are 16 and 8 Å in diameter, respectively. It is notable that the lining of each exit pore is dominated by lysine residues (figure 10*a*), conferring a positive charge on the opening and allowing the exit of negatively charged glutamic acid residues. A similar conformation of positively charged residues is observed in the exit pores of Sp PepA (figure 10*b*). By contrast, the exit pores of TET protease from *P. horikoshii* are dominated by hydrophobic phenylalanine residues (figure 10*c*), which may be explained by the fact that TET protease has a preference for leucine [27]. The positive surface potential of the MHJ_0125 small opening created by the lysine residues can be seen in figure 10*d*.

**Figure 11.**
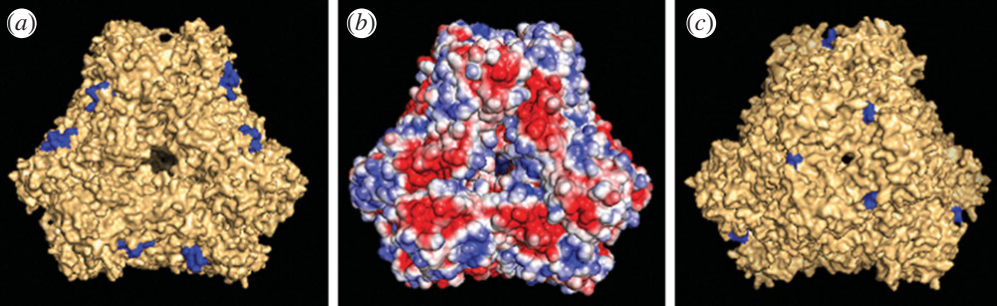
Surface rendering of the MHJ_0125 dodecameric structure showing the position of the heparin-binding consensus-like motif (*a*) and the C-terminal lysine residue likely to be involved in binding plasminogen (*c*). The surface potential of the dodecamer (rotated to look down the axis of the large pore) demonstrates that the heparin-binding motif (rich in lysine residues) confers a positive charge on these regions of the molecule's surface (*b*).

A motif (XBXBBX) similar to the consensus heparin-binding site (XBBXBX) was identified in the sequence of MHJ_0125. The EKMKKY motif is located at the N-terminus of MHJ_0125 and is exposed on the surface of the MHJ_0125 dodecamer (figure 11*a*). An XBXBBX motif has previously been implicated in the binding of heparan sulfate [28]. An examination of the surface potential of the MHJ_0125 dodecamer indicates that the area containing the XBXBBX motif is positively charged (figure 11*b*); therefore, these regions may play a role in interacting with the negatively charged sulfate groups of heparin.

The C-terminal lysine residues of each MHJ_0125 monomer are highlighted in figure 11*c*. C-terminal lysine residues have been implicated in the binding of several *M. hyopneumoniae* proteins to plasminogen [12,18]. These C-terminal lysine residues are exposed to the surface and are located in a small cleft created at the interface of the individual protein subunits.

## 4. Discussion

Little is known about the strategies used by mycoplasmas to secure essential protein components for life. As an obligate, genome-reduced pathogen, *M. hyopneumoniae* must derive amino acids from the host and transport them into the cell for the synthesis of essential proteins. Here, we describe the biochemical and structural features of a glutamyl aminopeptidase of the M42 family of aminopeptidases that displays the unusual capability to reside in the cytosol and on the surface of *M. hyopneumoniae* (figure 1). MHJ_0125 is hydrophilic, and lacks evidence of a signal sequence and transmembrane domains. rMHJ_0125 shows maximal aminopeptidase activity at physiological pH, requires divalent metal ions for activity and is inhibited by bestatin (figure 1). Consistent with MHJ_0125 molecules being located on the cell surface, freshly cultured *M. hyopneumoniae* cells displayed glutamyl aminopeptidase activity that was inhibited by the exogenous addition of bestatin. While MHJ_0125 molecules are both retained on the external side of the membrane and secreted into the extracellular milieu, the mechanism(s) that facilitate secretion of MHJ_0125 across the cell membrane is unknown.

MHJ_0125 forms a 440 kDa homopolymeric structure (figure 2) and was not detected as a monomer during native PAGE or size exclusion chromatography. Residues involved in metal ion binding (His^64^, Asp^179^, Glu^212^, Asp^234^ and His^320^) are well conserved among M42 aminopeptidases that belong to the TET family of dodecameric tetrahedral complexes [25,29]. Our modelling studies were consistent with this view. Asp^236^, Ser^238^, Leu^255^, Arg^257^, Thr^309^ and Gly^311^ have been shown to comprise the substrate-binding pocket in the M42 aminopeptidase PepA from *S. pneumoniae* [25]. While several of these amino acids are conserved in MHJ_0125, a notable omission is Arg^257^, which is purported to play a critical role in making electrostatic interactions with acidic N-terminus substrate residues (Asp/Glu) and Gly^311^. Modelling studies of MHJ_0125 show that a negatively charged aspartic acid residue in position 258 (Asp^258^) replaces a positively charged Arg^257^; however, it appears that the carboxyl moiety in Asp^258^ does not protrude sufficiently into the S1 pocket to confer negative charge to this region. Consistent with its role as a glutamyl aminopeptidase, the exit pore of MHJ_0125 is enriched in lysine residues that confer a positive charge to the pore opening and are likely to facilitate the expulsion of cleaved glutamyl residues (figure 10).

Proteins that reside in more than one cellular location typically are multifunctional and many belong to a remarkable subset of molecules known as moonlighting proteins [30]. Motifs needed to perform alternate functions are likely to be confined to regions of the molecule that do not disrupt the structural constraints required for MHJ_0125 to perform as a dodecameric glutamyl aminopeptidase. We show that in addition to aminopeptidase activity, rMHJ_0125 binds plasminogen and, in the presence of tPA, facilitates conversion of plasminogen to the active serine protease, plasmin. C-terminal lysine residues play an important role in binding plasminogen [12,18,31], and our modelling studies show that the C-terminal lysine of MHJ_0125 is accessible on the surface of the dodecamer. We propose that this surface-accessible lysine is involved in binding plasminogen. Several plasminogen-binding proteins have been described on the surface of *M. hyopneumoniae*, indicating that the capacity to recruit plasminogen to the cell surface is important during the normal course of infection [4,12,13,18]. Indeed, our *in vivo* studies are consistent with this view; plasminogen is found in close association with ciliated epithelial borders and plasmin activity is consistently higher in the BAL fluid of most pigs following challenge with *M. hyopneumoniae* [4].

In addition to binding plasminogen, we repeatedly observed MHJ_0125 to bind heparin. Recovery of a 440 kDa MHJ_0125 complex from heparin–agarose indicates that MHJ_0125 binds glycosaminoglycans in its native dodecameric conformation (figure 5). We identified a lysine-rich, putative heparin-binding motif (EKMKKY) that conforms to a known heparan sulfate-binding domain (XBXBBX) [28]. The motif was shown in our modelling studies to be accessible on the surface of MHJ_0125. *Mycoplasama hyopneumoniae* is highly dependent on interactions with glycosaminoglycans during colonization of respiratory tract cilia [21] and porcine epithelial cells [19], and the importance of this interaction is underscored by the degree of redundancy shown by members of the P97 and P102 paralogue families [11,13–16,18,19,21]. These adhesins promote close contact of *M. hyopneumoniae* with ciliated epithelium [1,2,5], where there is an abundance of glycosaminoglycans enriched in highly sulfated, heparin-like domains [32]. The secretion of MHJ_0125 from the surface of *M. hyopneumoniae* may play a role in colonizing new sites both in the respiratory tract and at distal tissue sites. In addition, the heparin-binding characteristics of MHJ_0125 may play a key role in targeting secreted MHJ_0125 and bound plasmin(ogen) to colonization sites on cilia and on the epithelial cell body in the porcine respiratory tract.

**Figure 12.**
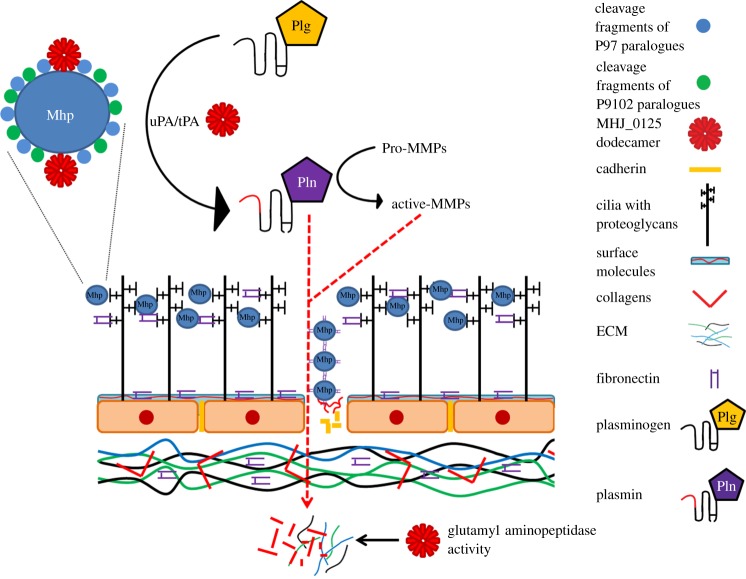
Interaction of *M. hyopneumoniae* with the porcine respiratory epithelium. P97 and P102 adhesin families bind glycosaminoglycans and fibronectin on the surface of porcine cilia. P97 and P102 adhesins and MHJ_0125, shown here as a dodecamer, bind porcine plasminogen and induce conformational changes that facilitate its conversion, on the *M. hyopneumoniae* cell surface, to plasmin by tPA and urokinase-type plasminogen activator (uPA). Plasmin is a broad spectrum serine protease that degrades host extracellular matrix (ECM) and other host proteins, and activates matrix metalloproteases (MMPs) that further breakdown ECM. Cilial and epithelial cell damage initiated by infection by *M. hyopneumoniae* stimulates the expression and secretion of fibronectin. The combined activity of plasmin and MMPs generates substrates for MHJ_0125 and other putative proteases on the surface of *M. hyopneumoniae*. Surface-bound plasmin targets cadherin located in intercellular junctions which presumably allows *M. hyopneumoniae* to gain entry to subepithelial sites.

Many pathogenic and environmental bacteria secrete hydrolytic enzymes into the extracellular milieu, where they influence adaption to their respective niche [33,34]. Bacterial extracellular proteases form part of an impressive arsenal to inflict significant tissue damage, destroy innate and adaptive immune effector molecules, modify the protein surface topography of both the organism that secretes them and the host (immune evasion), and provide nutrients for microbial growth [11,18,31,35,36]. Surface-accessible aminopeptidases work in concert with endoproteases to generate amino acids and peptides for nutrition and cell proliferation [36,37]. To our knowledge, this is the first report of an aminopeptidase that moonlights on the cell surface where it displays multifunctional binding properties, while retaining aminopeptidase activity. Recruitment of circulatory plasminogen onto bacterial cell surfaces by surface adhesins and its activation to plasmin underpins pathogenic processes used by many microbial agents [31]. Plasmin degrades tissue barriers by targeting heparan sulfate proteoglycans, laminin, type IV collagen and other key ECM components and is involved in the processing and subsequent activation of latent matrix metalloproteinases [31]. In light of our findings, we propose a model (figure 12) where cell surface MHJ_0125 participates in the recruitment of plasminogen to the surface of *M. hyopneumoniae* and assists in its activation to plasmin. The multitude of surface-accessible ECM-binding proteins derived from the P97 and P102 paralogue families recruit plasminogen to the cell surface where it degrades the ECM on epithelial surfaces and in the basal lamina of the respiratory tract and, potentially, at distal tissue sites. These digestion products provide a rich source of substrates for surface-accessible aminopeptidases such as MHJ_0125. This strategy is unlikely to be restricted to *M. hyopneumoniae* and may be widespread among microbial pathogens. Our study underscores the importance of investigating other surface-accessible moonlighting proteases, and presents new opportunities for designing therapeutic compounds to control microbial pathogens (figure 12).

## 5. Material and methods

### 5.1. Ethics statement

Animal procedures were approved by the Animal Ethics Committee at the Elizabeth Macarthur Agricultural Institute (AEC project number M08/12) and were in accordance with the *Australian Code of Practice for the Care and Use of Animals for Scientific Purposes*.

### 5.2. Materials

H-Glu-AMC, H-Asp-AMC, H-Arg-AMC, H-Pro-AMC, H-Leu-AMC, H-Val-AMC, H-Ala-AMC and H-Phe-AMC were purchased from Bachem (St. Helens, UK). Bestatin, EDTA, trypsin, tributylphosphine, acrylamide, ExtrAvidin peroxidase and 3,3-diaminobenzidine were obtained from Sigma (Sydney, Australia). Pre-cast gels, buffers and molecular weight markers as well as standard molecular biology reagents were purchased from Life Technologies (Australia) unless stated otherwise.

### 5.3. *Mycoplasma* culture conditions

*Mycoplasma hyopneumoniae* (strain J) was grown in modified Friis broth [38]. Cells were harvested by centrifugation at 10 000×*g* and pellets were stored at −20°C until use as described previously [39].

### 5.4. High-resolution clear native PAGE and two-dimensional high-resolution clear native/SDS-PAGE

Freshly harvested *M. hyopneumoniae* cells were washed extensively (more than three times) in PBS and pelleted by centrifugation (9000×*g* for 10 mins at 4°C). The resulting 0.1 g cell pellet was resuspended in 1 ml 1 × native sample buffer supplemented with 1 per cent *n*-dodecyl β-d-Maltoside and 1 × complete protease inhibitor cocktail (Roche) and solubilized for 30 min on ice. Insoluble material was removed by centrifugation at 4°C and soluble proteins were desalted using a MicroBiospin column (BioRad). Samples were run on 4–16% NativePAGE gels at 4°C using high-resolution clear native electrophoresis with cathode buffer supplemented with sodium deoxycholate only [40]. One-dimensional gels were fixed with 40 per cent methanol/10 per cent acetic acid for 30 min, stained with colloidal Coomassie blue overnight before destaining with 1 per cent acetic acid. Alternatively, lanes for denaturing two-dimensional gel electrophoresis were excised from the gel and equilibrated in SDS equilibration solution (2% SDS, 6 M urea, 250 mM Tris–HCl pH 8.5, 0.0025% (w/v) bromophenol blue) for 20 min before the second dimension SDS-PAGE. The resulting gels were fixed and stained as above or stained with Flamingo fluorescent stain (BioRad) for 1–2 h and visualized using a Pharos FX-Plus gel scanner (BioRad).

### 5.5. Detection and isolation of *Mycoplasma hyopneumoniae* surface-expressed and -secreted proteins

Methods used to biotinylate and recover surface proteins labelled with biotin and generate tryptic peptides of surface-exposed proteins (surface shaving) and characterize them by LC-MS/MS have been described previously [11,16,18]. Procedures used to detect surface-accessible proteins by graded trypsin hydrolysis and western blotting have also been described previously [17,41]. Secreted proteins were recovered from control shaving experiments. Freshly cultured *M. hyopneumoniae* cells were harvested by centrifugation, washed twice with PBS and resuspended in PBS pH 7.4 at 37°C for 5 min (no added trypsin). *Mycoplasma hyopneumoniae* cells were removed by centrifugation and proteins released into the PBS during this 5 min incubation at 37°C (secreted protein fraction) were retained in the supernatant. Their identities were determined by trypsin digestion followed by LC-MS/MS as described previously [11,18].

### 5.6. Proteomics

The preparation of peptides for mass spectrometry analysis has been described in detail previously [11,42]. Briefly, excised gel spots/bands were reduced and alkylated with 5 mM tributylphosphine and 20 mM acrylamide for 90 min at room temperature. The gel pieces were then digested with 12.5 ng ml^−1^ sequencing grade trypsin at 37°C overnight and the resulting peptides were solubilized with 2 per cent formic acid (v/v) prior to analysis by LC-MS/MS using a Tempo nanoLC system (Applied Biosystems) with a ProteoPep II C18 column (New Objective, Woburn, MA) coupled to a QSTAR Elite hybrid QTOF mass spectrometer (Applied Biosystems/MDS Sciex). An intelligent data acquisition experiment was performed, with a mass range of 375–1500 Da continuously scanned for peptides of charge state 2^+^–5^+^ with an intensity of more than 30 counts s^−1^. Selected peptides were fragmented and the product ion fragment masses measured over a mass range of 100–1500 Da. The mass of the precursor peptide was then excluded for 120 s. The MS/MS data files were searched using Mascot (v. 2.2.2, provided by the Australian Proteomics Computational Facility, http://www.apcf.edu.au/) against the LudwigNR database v. Q211 which is composed of the UniProt, plasmoDB and Ensembl databases (8 785 680 sequences, 3 087 386 706 residues). The enzyme specificity was set to trypsin with three missed cleavages allowed. Propionamide and oxidation of methionine were set as variable protein modifications, while no fixed modifications were set. The mass tolerance was set at 100 ppm for precursor ions and 0.2 Da for fragment ions.

### 5.7. Site-directed mutagenesis

Since *Mycoplasma* species use the UGA codon to encode tryptophan rather than signalling the end of translation, the expression of *Mycoplasma* proteins in *E. coli* results in the production of truncated recombinants. Therefore, site-directed mutagenesis (by fusion PCR) was used to remove premature stop codons from the MHJ_0125 gene (Genbank accession AAZ44217.1). Primers extending in opposite directions were designed across the target mutagenesis site (MutF125: 5′-GAATTTG**G**GGAAATGCGGTAATAG-3′ and MutR125: 5′-TTCCCCAAATTCCGCCAATTG-3′) with 12 base overlap (underlined) and including an A to G transition to convert a premature stop codon (TGA) into tryptophan (TGG). The two-step fusion PCR was done initially by pairing the mutagenesis primers with primers designed to anneal at the two boundaries of the target gene (F125: 5′-CACCATGTCAATATTAGAAAAAATGAAAA-3′, R125: 5′-CTTTAATATCTGATAAACTAATTAGACCATAAG-3′), designed following instructions in the Champion pET directional TOPO Expression kit. The two separate PCR products were then fused in a seven-cycle PCR reaction, followed by extension of the fused products with boundary primers to generate the insert for the pET expression system. All PCR reactions were performed using the Platinum *Pfx* DNA polymerase proof-reading enzyme. The mutagenized MHJ_0125 template DNA was cloned into the pET100/D-TOPO expression vector (Life Technologies), and following DNA sequencing (Macquarie University Sequencing Facility), the construct was transformed into BL21 (DE3) *E. coli* for expression.

### 5.8. Expression and purification of recombinant MHJ_0125 in *Escherichia coli*

Soluble His-tagged rMHJ_0125 protein was purified by affinity chromatography using Profinity IMAC Ni-charged resin (BioRad) according to the manufacturer's instructions. Briefly, the cleared lysate was loaded onto a 1.2 ml (50% slurry) IMAC column and washed with six column volumes of wash buffer (300 mM KCl, 50 mM KH_2_PO_4_, 20 mM imidazole). The rMHJ_0125 protein was eluted from the column in the same buffer containing 250 mM imidazole, dialysed into PBS (pH 7.3) and stored in aliquots at −20°C until use.

### 5.9. Enzymatic analysis

The aminopeptidase activity of rMHJ_0125 was determined by measuring the release of the fluorogenic leaving group, AMC, from a range of fluorogenic peptide substrates representative of the various amino acid groupings as previously described [43]. Reactions were carried out in 96-well microtitre plates (200 μl total volume, 1 h, 37°C) using a spectrofluorometer (Bio-Tek KC4) with excitation at 360 nm and emission at 460 nm. Generally, purified recombinant enzyme (30 nM), or cultured *M. hyopneumoniae* cells (1 × 10^5^ cells/well), were incubated in 50 mM Tris–HCl (pH 7.5) containing 5 mM CoCl_2_ for 20 min before the addition of 50 μM substrate. Assays were also performed in a range of 50 mM buffers: sodium acetate (pH 4.5–5.5), sodium phosphate (pH 5.5–8.0) and sodium borate (pH 8.0–9.0), and in the presence of a range of 5 mM metal chlorides (CoCl_2_, CuCl_2_, MnCl_2_, MgCl_2_ and ZnCl_2_). The susceptibility of MHJ_0125 to the aminopeptidase inhibitor bestatin and to the metal ion chelator EDTA was determined by performing assays against H-Glu-AMC following pre-incubation (10 min at 37°C) with each over the range 0.01–1 mM.

### 5.10. Porcine plasminogen purification

Plasminogen was isolated from porcine plasma as described previously [12,13]. The isolated protein was confirmed as plasminogen following LC-MS/MS analysis. Biotinylation of plasminogen for ligand blot analysis was performed using Sulfo-NHS-LC-Biotin (sulfosuccinimidobiotin) according to the manufacturer's instructions. To remove excess biotin the biotinylated plasminogen was dialysed against PBS at 4°C.

### 5.11. Porcine plasminogen activation assay

The approach used to test whether rMHJ_0125 was able to enhance activation of plasminogen is described in detail elsewhere [4]. Porcine plasminogen (50 µg) was pre-incubated for 1 h at 37°C with 1 : 1, 1 : 4 and 1 : 8 molar ratios of MHJ_0125 in microtitre plate wells (Greiener Bio One, Frickenhausen, Germany) for these assays.

### 5.12. Western and ligand blot analysis

Blots of lysates from *M. hyopneumoniae* cells exposed to different concentrations of trypsin in the range 0–500 µg ml^−1^ were separately probed with rabbit anti-P97 N-terminal serum (1 : 100) as described previously [15,17], and rabbit anti-MHJ_0125 serum (1 : 100) followed by goat anti-rabbit HRP conjugate (1 : 1000). In control experiments, blots probed only with goat anti-rabbit conjugate (1 : 1000) did not produce any detectable bands. The binding of rMHJ_0125 to porcine plasminogen was investigated using ligand blotting. Serially diluted rMHJ_0125 samples were run on 4–12% Criterion Bis–Tris gels and transferred to PVDF membranes at 300 mA for 30 min. Blots were blocked with 5 per cent non-fat dry milk in PBS Tween 20 (0.1% v/v). The membranes were then incubated with a 1 : 250 dilution of biotinylated porcine plasminogen for 1 h at room temperature followed by ExtraAvidin peroxidase (1 : 3000) for 1 h. To determine the immunogenicity of MHJ_0125, blots were probed with a pool of convalescent pig sera (1 : 50) collected during *M. hyopneumoniae* infection and detected with peroxidase-conjugated anti-pig IgG (diluted 1 : 5000). Both ligand and Western blots were visualized by the addition of 3,3-diaminobenzidine peroxidase substrate.

### 5.13. Generation of rabbit sera against MHJ_0125

Monospecific, polyclonal antibodies against rMHJ_0125 were generated in New Zealand White rabbits using a protocol described previously [41]. All experiments involving animals were conducted with animal ethics approval.

### 5.14. Fluorescence microscopy

Glass coverslips (13 mm, no. 1.5 thickness; Gerhard Menzel GmbH, Braunschweig, Germany) were coated in 0.01% poly-l-lysine for 10 min, dried at 55°C for 1 h and placed in a 24-well microtitre plate. One ml of *M. hyopneumoniae* strain J culture was centrifuged (10 000×*g* for 10 min) and washed three times with 1 ml sterile PBS. Washed cells (200 µl) were added to wells containing glass coverslips and allowed to adhere at 37°C for 30 min. Wells were washed once with PBS and fixed in 4 per cent paraformaldehyde at 4°C for 30 min. To permeabilize cells, 0.1 per cent Triton X-100 in PBS was added for 5 min and washed three times with PBS. To quench excess aldehydes, 100 mM glycine in PBS was added for 5 min and washed three times with PBS. Non-specific binding sites were blocked in 2 per cent bovine serum albumin (BSA) in PBS overnight at 4°C and washed three times in PBS. Rabbit MHJ_0125 antisera and control rabbit antisera were each diluted 1 : 100 with PBS containing 1 per cent BSA, incubated for 1 h at room temperature and washed three times in PBS. A 1 : 1000 dilution of goat anti-rabbit antibodies conjugated to Alexa Fluor 488 (Life Technologies) was prepared in PBS containing 1 per cent BSA and incubated for 1 h at room temperature and washed three times in PBS. Coverslips were mounted onto microscope slides in 2 μl of VECTASHIELD, sealed using nail varnish and imaged on an Olympus BX51 Upright Epi Fluorescence microscope. Images were captured by an Olympus DP70 Digital Microscope Camera using Olympus DP Controller software with manual intensity exposure settings at 9–10. These same slides were imaged using super-resolution 3D-SIM using a DeltaVision OMX Imaging System (Applied Precision Inc., Issaquah, WA, USA) as previously described [44].

### 5.15. Size exclusion chromatography

Gel filtration chromatography was carried out by loading 10 µl of purified rMHJ_0125 protein (10 µg) onto a Superdex 200 column (3.2 mm ID × 300 mm) equilibrated with Tris–HCl (20 mM, pH 8.0). Chromatography was performed with a flow rate of 50 µl min^−1^ using a Waters Alliance 2690 HPLC system, controlled by MassLynx v. 4.0. Eluted proteins were detected with a Model 990 PDA UV detector by monitoring absorbance at 220 and 280 nm.

### 5.16. Heparin column chromatography

*Mycoplasma hyopneumoniae* cells were lysed in 10 mM phosphate buffer by sonication on ice for 3 × 15 s bursts at 50 per cent power and insoluble material was pelleted by centrifugation. A 1 ml HiTrap heparin column was equilibrated with 10 mM phosphate buffer and the sample was loaded at 0.5 ml min^−1^ over 10 min, then washed with 10 mM phosphate buffer for a further 10 min to remove non-specific binders before running an increasing linear gradient to 1 M NaCl over 25 min then to 2 M NaCl over 10 min and maintained for 5 min to elute any remaining protein from the column before returning to 10 mM phosphate buffer over 5 min and re-equilibrating. The chromatographic profile (A_280_) indicated all non-heparin-binding proteins were collected in the first 15 min. Heparin-binding proteins started to elute at approximately 35 min at a NaCl concentration of about 300 mM. Elutions were collected over 20 min and pooled for analysis. SDS-PAGE was also used to monitor the elution profile of proteins from the heparin column. Pooled elutions were analysed by native PAGE to determine the presence of complexes. Heparin-binding proteins, and eluted complexes, were identified by LC-MS/MS using standard procedures [15].

### 5.17. Bioinformatics and phylogenetic analysis

MHJ_0125 orthologues in other *Mycoplasma* strains and bacterial species, as well as related sequences from metazoans, were identified following BLASTp analyses of the UniProt Knowledgebase (http://web.expasy.org/blast/) using the MHJ_0125 primary sequence as search query. Selected sequences were aligned using Clustal W and Espript [45,46]. Phylogenetic trees were created using 33 *Mycoplasma* and bacterial amino acid sequences and six metazoan sequences. The protein sequences were initially aligned using Clustal W [45] and the trees were created using the bootstrapped (1000 trials) neighbour-joining method of MEGA v. 4.0 [47], using the Kimura two-parameter model with uniform rates for all sites. The UniProt accession numbers of the sequences used for alignment and phylogenetic analyses can be found in electronic supplementary material, figure S5.

### 5.18. Modelling of MHJ_0125

The three-dimensional structure of MHJ_0125 was reconstructed using comparative modelling. BLAST analysis was used to identify a number of related structures within the PDB, which were considered as potential templates, and the program Modeller [48] was used to select the most suitable template for construction of the model. The align2d function in Modeller was used to align MHJ_0125 to TET protease from *P. horikoshii* (PDB ID: 1Y0R) according to secondary structure. For comparative modelling, the *P. horikoshii* TET protease dodecamer was assembled using the BioMT figures provided in the PDB file. Modeller was then used to generate the 3-dimensional structure of the MHJ_0125 using the TET protease dodecamer as a template. *Pyrococcus horikoshii* TET protease residues V115–K127 were not determined during crystallography owing to the fact that these residues are buried, therefore the corresponding residues (T116–K128) were excluded from the MHJ_0125 dodecameric model. The quality of the model was checked with ProSA [49,50]. The final structure of MHJ_0125 was rendered using the PyMOL Molecular Graphics System, v. 1.5.0.4, Schrödinger, LLC. To determine the electrostatic potential of the MHJ_0125 surface, Poisson Boltzmann electrostatics calculations were performed using PDB2PQR [51,52] and were rendered in PyMOL using the Adaptive Poisson Boltzmann software plugin [53].

## 6. Acknowledgements

We are grateful for the assistance of G. J Eamens for the preparation of anti-MHJ_0125 serum in rabbits. This work was supported by a UTS start up grant to S.P.D. J.L.T. and B.B.R. are recipients of PhD scholarships from UTS. C.B.W. was supported by an Australian NHMRC Senior Research Fellowship (571905).

## Supplementary Material

Figure S1

## Supplementary Material

Figure S2

## Supplementary Material

Figure S3

## Supplementary Material

Figure S4

## Supplementary Material

Figure S5
