# Supplementary figures and images for: MHJ_0125 is an M42 glutamyl aminopeptidase that moonlights as a multifunctional adhesin on the surface of Mycoplasma hyopneumoniae
Source: Open Biol. 2013 Apr;3(4):130017. doi: 10.1098/rsob.130017 (PMC3718333; doi:10.1098/rsob.130017)

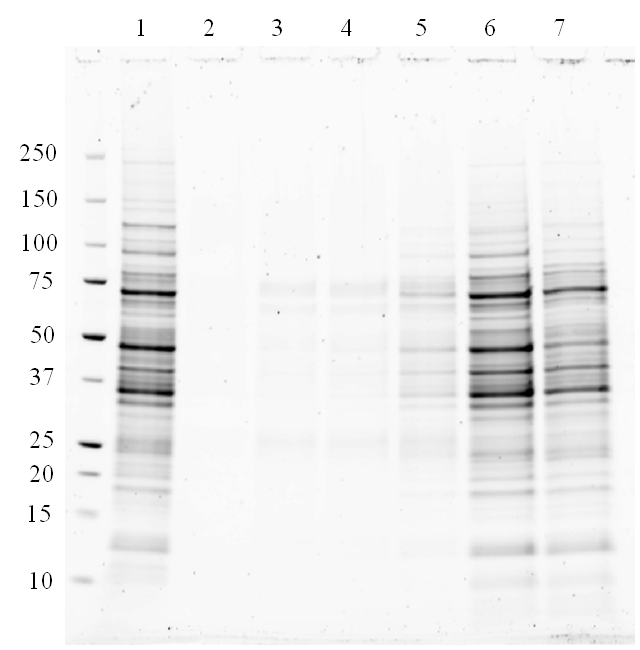

Supplement: Figure S1 [file rsob130017-s1.tif]

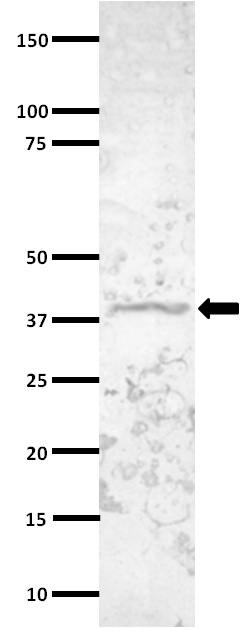

Supplement: Figure S2 [file rsob130017-s2.tif]

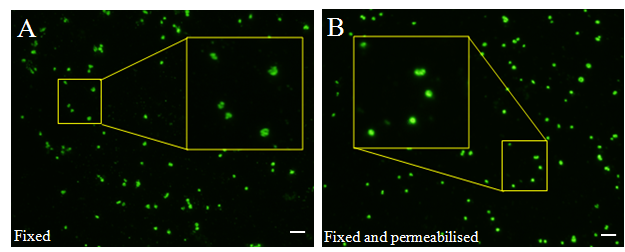

Supplement: Figure S3 [file rsob130017-s3.TIF]

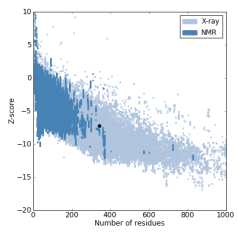

Supplement: Figure S4 [file rsob130017-s4.tif]

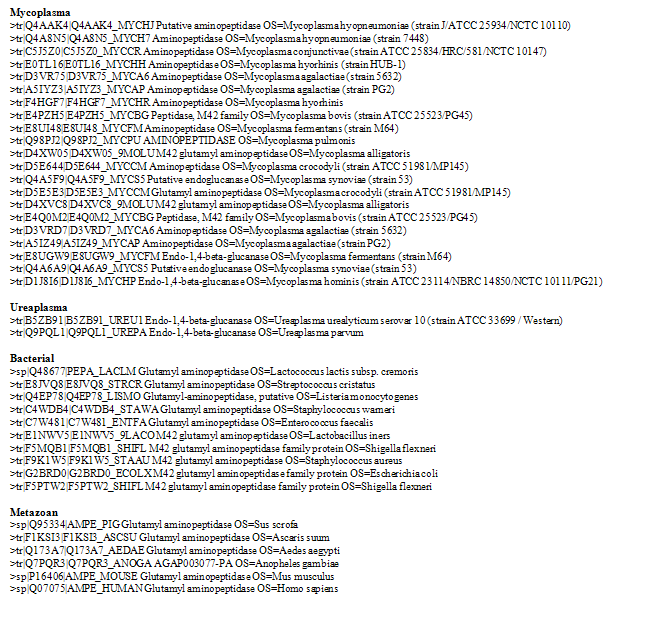

Supplement: Figure S5 [file rsob130017-s5.tif]
